# *Plasmodium knowlesi* malaria an emerging public health problem in Hulu Selangor, Selangor, Malaysia (2009–2013): epidemiologic and entomologic analysis

**DOI:** 10.1186/1756-3305-7-436

**Published:** 2014-09-15

**Authors:** Indra Vythilingam, Yvonne AL Lim, Balan Venugopalan, Romano Ngui, Cherng Shii Leong, Meng Li Wong, LokeTim Khaw, XiangTing Goh, NanJiun Yap, Wan Yusoff Wan Sulaiman, John Jeffery, Ab Ghani CT Zawiah, Ismail Nor Aszlina, Reuben SK Sharma, Lau Yee Ling, Rohela Mahmud

**Affiliations:** Department of Parasitology, Faculty of Medicine, University of Malaya, Kuala Lumpur, Malaysia; State Vector Borne Disease Control Unit, Selangor State Health Department, Selangor, Malaysia; Vector Control Unit, Selangor Health Department, Block C, Jalan Langat, Bandar Botanik, Klang, Malaysia; Hulu Selangor Health District, Kuala Kubu Bahru, Selangor, Malaysia; Department of Veterinary Laboratory Diagnosis, Faculty of Veterinary Medicine, Universiti Putra Malaysia, 43400 UPM Serdang Selangor, Malaysia

**Keywords:** *An. introlatus*, Vector, *P. knowlesi*, Malaysia

## Abstract

**Background:**

While transmission of the human *Plasmodium* species has declined, a significant increase in *Plasmodium knowlesi/Plasmodium malariae* cases was reported in Hulu Selangor, Selangor, Malaysia. Thus, a study was undertaken to determine the epidemiology and the vectors involved in the transmission of knowlesi malaria.

**Methods:**

Cases of knowlesi/malariae malaria in the Hulu Selangor district were retrospectively reviewed and analyzed from 2009 to 2013. Mosquitoes were collected from areas where cases occurred in order to determine the vectors. Leucosphyrus group of mosquitoes were genetically characterized targeting the nuclear internal transcribed spacer 2 (ITS2) and mitochondrial cytochrome c oxidase subunit I (CO1). In addition, temporal and spatial analyses were carried out for human cases and vectors.

**Results:**

Of the 100 microscopy diagnosed *P. knowlesi/P. malariae* cases over the 5 year period in the Hulu Selangor district, there was predominance of *P. knowlesi/P. malariae* cases among the young adults (ages 20–39 years; 67 cases; 67%). The majority of the infected people were involved in occupations related to agriculture and forestry (51; 51%). No death was recorded in all these cases.

Five hundred and thirty five mosquitoes belonging to 14 species were obtained during the study. *Anopheles maculatus* was the predominant species (49.5%) followed by *Anopheles letifer* (13.1%) and *Anopheles introlatus* (11.6%)*.* Molecular and phylogenetic analysis confirmed the species of the Leucosphyrus group to be *An. introlatus*. In the present study, only *An. introlatus* was positive for oocysts. Kernel Density analysis showed that *P. knowlesi* hotspot areas overlapped with areas where the infected *An. introlatus* was discovered. This further strengthens the hypothesis that *An. introlatus*is is the vector for *P. knowlesi* in the Hulu Selangor district.

Unless more information is obtained on the vectors as well as macaque involved in the transmission, it will be difficult to plan effective control strategies. The utilization of modern analytical tools such as GIS (Geographic Information System) is crucial in estimating hotspot areas for targeted control strategies.

**Conclusions:**

*Anopheles introlatus* has been incriminated as vector of *P. knowlesi* in Hulu Selangor. The cases of *P. knowlesi* are on the increase and further research using molecular techniques is needed.

## Background

Malaria still remains a serious public health challenge in the tropics. However, with improved and effective treatment coupled with the use of long lasting insecticide treated nets, the burden of disease has decreased over the years [1]. In highly endemic countries, this disease is observed to be more preponderant in infants and young children [2]. Lately, with the decline in the number of cases, a contrasting observation noted that older children and adults are more susceptible to the disease.

Historically, malaria used to be a major health concern in Malaysia with >200,000 cases in the 1960s [3]. The introduction of a malaria eradication programme in the late 1960s, which continued to the late 80s has contributed to the significant reduction of malaria cases in Malaysia to approximately 43,000 cases in the early 1990s. During those years, the states of Kelantan, Pahang and Perak had the highest number of cases in peninsular Malaysia [4]. During the eradication period, DDT residual house spraying was the main method used to reduce malaria vectors. With economic development and the introduction of insecticide treated bednets, malaria was further reduced to approximately 12,000 cases in 2000. In 2001, 12,780 cases were reported and this was significantly scaled down to 4,725 cases in 2012 [5].

Four species of malaria parasites (namely *Plasmodium falciparum, P. vivax, P. malariae* and *P.ovale*) are known to affect humans. In 2004, Singh *et al.* [6] reported the presence of a large number of *P. knowlesi* cases in Sarawak, a state in Malaysian Borneo. Subsequently, *P. knowlesi* became known as the fifth malaria parasite species to infect humans [7]. Various studies have shown that *P. knowlesi* is not only present in Malaysia but has a wider presence in other parts of Southeast Asia as well, namely Singapore [8–10], Thailand [11–14], Myanmar [15, 16], Philippines [17], Indonesia [18, 19], Vietnam [20, 21] and Cambodia [22]. To date, only Laos and Timor-Leste are devoid of any reported *P. knowlesi* cases.

Lately, it was observed that as transmission of the human *Plasmodium* species declined, a significant increase in microscopy diagnosed *P. knowlesi/P. malariae* cases were reported [23]. A similar trend was also observed in peninsular Malaysia [24]. This was confirmed in the Ministry of Health Annual Report that in 2012, *P. knowlesi* was the predominant species comprising 38% of the malaria cases in Malaysia followed by *P. vivax* (31% of cases) [5]. In Malaysian Borneo, Sabah and Sarawak contributed most of the cases, whereas Pahang has always had a high number of *P. knowlesi* malaria cases [24, 25] in peninsular Malaysia.

Malaria is transmitted from one person to another by the bite of the female infected *Anophele*s mosquito. The main malaria vectors in Malaysia are *Anopheles campestris* and *An. sundaicus* (now known as *An. epiroticus*) in the coastal areas, and *An. maculatus* in the hilly regions of peninsular Malaysia, *An. latens* and *An. balabacensis* the predominant vectors in Sarawak and Sabah, respectively [26–28]. The malaria eradication programme worked very well in the coastal areas and the vectors were almost eliminated due to their resting behavior indoors [29]. However, in the hilly regions of peninsular Malaysia, *An. maculatus* remains the primary vector of human malaria. When the first case of *P. knowlesi* infection was reported in Pahang, Malaysia, the Leucosphyrus group of mosquitoes was incriminated as the vectors. The vectors incriminated were *An. hackeri* (for *P. knowlesi*) in the coastal area of Selangor [30] and *An. cracens* (then known as *An. balabacensis*) in Perlis (northern region of peninsular Malaysia) (for *Plasmodium inui)* [31]. However, *An. hackeri* was found to only biting monkeys, and was not reported to bite humans. In 2006, *An. latens* was incriminated as a vector *of P. knowlesi* in Kapit Sarawak [32, 33], *An. cracens* in Kuala Lipis Pahang [25, 34] and *An. balabacensis* in Sabah [35].

The state of Selangor consists of nine districts and the Hulu Selangor district had the highest number of knowlesi/malariae malaria cases (Records from State Health Department). For the past five years, the cases of knowlesi/malariae malaria have also increased in Hulu Selangor. However, the vector involved in the transmission of knowlesi malaria remained unknown in this district. The national malaria program strategy has been reoriented from control to elimination, and Malaysia is now working to eliminate malaria from the peninsular by 2015 and from Malaysian Borneo by 2020 [36].

In view of these recent events, it is imperative that a holistic approach is adopted in order to understand the transmission of *P. knowlesi* malaria better especially in this district. Thus, the objective of this study was to analyse the epidemiology of knowlesi/malariae malaria in Hulu Selangor in a more comprehensive manner, taking into consideration the human infection and vector distribution. This study actively collected mosquitoes from locations with human cases and in so doing, managed to determine the vectors in Hulu Selangor. Another interesting feature of the study involved the temporal and spatial analysis utilizing advanced tools such as GIS analysis to shed some light on the association of human infection and mosquito distribution in order to elucidate the transmission of *P. knowlesi* malaria in Hulu Selangor.

## Methods

### Ethical clearance

This study was approved by the Medical Ethics Committee of University of Malaya Medical Center (UMMC), Malaysia (Reference number: 890.12) and the National Medical Research Register (Reference number: NMRR-11-1050-10619). Prior to mosquito collection, volunteers who consented were provided antimalarial prophylaxis.

### Epidemiology aspects

#### Study site

This study was carried out in the state of Selangor, the most developed state [23.5% of Malaysia’s total GDP (gross domestic product) in 2012; Department of Statistics, Malaysia] among the 13 states in Malaysia equipped with modern infrastructure. The state also has the most concentrated population in Malaysia, with high living standards and the lowest poverty rate in the country. Selangor state consists of 9 districts, namely, Gombak, Hulu Langat, Hulu Selangor, Klang, Kuala Langat, Kuala Selangor, Petaling, Sabak Bernam and Sepang.

For the present study, the focus area was on the Hulu Selangor district which is located in the northeastern part of Selangor. Its principal town is Kuala Kubu Bharu (KKB) (3°34′N 101°39′E / 3.567°N 101.650°E). The district of Hulu Selangor is blessed with natural beauty such as lush greenery, hilly terrains, scenic lakes and waterfalls. It provides an ideal place for nature activities especially for the city dwellers. The local people are mostly involved in agricultural activities, small businesses and factories. There are also communities of the indigenous people (locally known as the ‘Orang Asli’) living in this district.

### Retrospective review of human malaria cases

Records of all human cases positive for *P.knowlesi/P. malariae* in the district of Hulu Selangor were meticulously retrieved from 1 January 2009 till 31 December 2013 from Kuala Kubu Bharu Health office in the district and cross-checked with data from the State Health Department, Selangor. These cases were microscopy diagnosed by experienced laboratory technicians and all positive cases and 10% of the negative cases were examined by the State Health Department, Selangor. Due to the limitatations of microscopy in distinguishing *P. knowlesi* and *P. malariae* species, it was decided to consider both these species as a single group (*P. knowlesi/P. malariae*) as practiced by William *et al.* [23] in their study.

Information regarding age, gender, race/nationality, occupation, work address, home address, location of source of infection (obtained during case investigation exercise) coupled with its latitudinal and longitudinal coordinates were gathered and entered into a database. To reduce any data entry error, entries were doubled checked by at least two data entry assistants.

### Statistical analysis

Data analysis was carried out using the Statistical Package for the Social Sciences (SPSS) for Windows version 17 (SPSS Inc, Chicago, USA). Categorical variables such as infection rate was presented as frequencies and percentages.

### Entomology

The study was carried out in the Hulu Selangor district from 2012 to 2013. Various locations were surveyed within the district where knowlesi malaria cases were reported. The sites surveyed were possible locations where the cases could have picked up the infection. Most of the sites were in secondary forested areas or in rubber plantations surrounded by scrub forest. The study sites included lowland areas and hilly terrains.

### Mosquito collection, identification and dissection

Mosquito collections, using the bare-leg catch method [37] was carried out from 19:00 to 00:00. This is the only proven method of collection of *Anopheles* mosquitoes in this country. All mosquitoes collected were brought back to the laboratory for identification. The keys of Reid [26] and Sallum [38] were used for the identification of *Anopheles* mosquitoes. All *Anopheles* mosquitoes were dissected to extract the midguts, salivary glands and ovaries and these were examined for oocysts and sporozoites and parity status respectively.

### DNA extraction and PCR

DNA was extracted from positive midguts and Leucosphyrus group mosquitoes using the DNeasy tissue kit (Qiagen, Germany), according to the manufacturer’s protocol. The extracted DNA was kept at -20°C until required. A hexaplex PCR detection system was employed to simultaneously detect the various *Plasmodium* species present according to a published protocol [39].

### Molecular identification and sequencing targeting the internal transcribed spacer 2 (ITS2) and cytochrome c oxidase subunit I (CO1)mt DNA

To confirm the species of *An. introlatus*, PCR was carried on targeted at ITS2 and CO1 genes. The ITS2 was amplified using primers ITS2A (5′TGTGAACTGCAGGACA 3′) and ITS2B(5′TATGCTTAAATTCAGGGGGT 3′) [40]. For CO1, the primers used were UEA9.2 (5′CTA ACA TTT TTT CCT CAA CAT TTT TTA GG-3′) and UEAlO.2 (5′TTA TTAGTT AAT AAY GGT ART TCT G-3′) [41]. PCR reactions were performed as previously described [41] without modifications. Amplicons were subjected to electrophoresis on 1.5% agarose gels (Promega, Madison, WI). The amplified product from the gel was purified and sequenced bidirectionally on an ABI prism sequencer using the PCR primers (Genomics Bioscience and Technology Co Ltd. China).

### Analysis of sequence data

Sequences were aligned and checked manually using BioEdit (version 7.1.11) (Applied Biosystem, UK). The sequences were aligned with other representative sequences obtained from the GenBank using ClustalW version 1.7. A Neighbor Joining (NJ) phylogenetic tree was constructed using the MEGA version 5.1 software with 1000 bootstrap replicates. Branches corresponding to partitions reproduced in less than 80% of bootstrap replicates were collapsed. All sequences were submitted to GenBank(KM032605;KM032606;KM032607;KM032608;KM032609;KM032610;KM032611;KM032612;KM032613;KM032614;KM032615;KM032616;KM032617;KM032618;KM032619;KM032620;KM032621).

### Geo-positioning, temporal and spatial analysis

The geographic coordinate for *P. knowlesi/P. malariae* human cases from 2009 to 2013 with complete addresses of the source of infection were determined using handheld Garmin GPSMAP 60CSx and downloaded from the GPS memory card into the computer using the GPS Pathfinder software. These geographic coordinates were synchronized using World Geodetic System (WGS 1984), which serve the x (longitude or east–west) and y (latitude or north–south). The recorded coordinates of each location were imported into Microsoft Excel and documented as a vector program database. All the geo-positioned cases were then exported and stored into ArcGIS 9.3 software (ERSI, Redlands, CA, USA) as raster format for further spatial risk patterns analyses and exploration. These were then geo-referenced on a GIS map layer at a Hulu Selangor sub-district level as point features to create a new GIS layer representing the point location of each *P. knowlesi/P. malariae* case. The collected *An. introlatus* mosquitoes were also geo-referenced in the same layer as *P. knowlesi*/*P. malariae* cases to create a GIS database format.

The spatial distribution of *P. knowlesi* cases was analyzed using the Average Nearest Neighbor (ANN) method, a method that provides indication of whether the spatial distribution pattern of particular a case occurs randomly, is clustered or dispersed [42]. The ANN method measures the average nearest neighbor index (R) value of the particular distance between each feature location and its nearest neighbour’s location. If the R value is less than 1 (R < 1), the distribution of the cases being analyzed are considered clustered. If the R value is greater 1 (R > 1), the distribution patterns are considered dispersed, while if the R value is to equal 1 (R = 1), it indicates a random distribution pattern. The Z value indicates if the observed pattern is significantly different from a random pattern with a significance level of 0.001 [42]. The Average Nearest Neighbor (ANN) ratio was calculated as:


Where, ***D***_***o***_ is the observed average distance between each case and its nearest neighbours’ cases and ***D***_***E***_ is the expected average distance for the cases given in a random pattern.

A hot spot analysis using the Kernel Density estimation and interpolating technique was carried out to identify high-risk areas and density of *P. knowlesi/P. malariae* cases in neighborhood locations with reported infections. Kernel density estimation is an effective tool to identify high-risk areas within point patterns of disease incidence by producing a smooth, continuous surface that defines the level of risk for that area [43, 44]. The interpolation and dissemination pattern of *P. knowlesi*/*P. malariae* cases using Kernel Density is estimated based on the given formula:


Where, ***λ (s)*** is the estimated infected value by *P. knowlesi* per area; **г** is the smoothing factor, ***k*** is the kernel weighting function and *s* is the centre of the area and ***si*** is the location of the case.

## Results

### Overall malaria cases from 2009–2013 in Selangor state

From 2009 until 2013, there were a total of 1,284 cases of malaria reported in the state of Selangor, Malaysia. Of this total, 915 (71.3%) were caused by *P.vivax*, 175 (13.6%) by *P. falciparum*, 171(13.3%) by *P. knowlesi/P. malariae,* 22 (1.7%) mixed infections and 1 (0.1%) by *P. ovale* (Table 1)*.* Based on the total samples according to year, there were 231 cases in 2009 with number of cases declining to 196 in 2010 before hovering around 286 (2011), 270 (2012) and 301 (2013) in recent years. However, only about 30% (379) of cases were locally acquired.

**Table 1:** **Malaria cases according to species from 2009 till 2013 for the state of Selangor**

Parasite species	2009	2010	2011	2012	2013	Total
***P. vivax***	179 (77.5%)	131 (66.8%)	193 (67.5%)	199 (73.7%)	213 (70.8%)	915 (71.3%)
***P. falciparum***	31 (13.4%)	28 (14.3%)	42 (14.7%)	28 (10.4%)	46 (15.3%)	175 (13.6%)
***P. knowlesi /P. malariae***	21 (9.1%)	25 (12.8%)	43 (15%)	41 (15.2%)	41 (13.6%)	171 (13.3%)
**Mixed infections**	0	11 (5.6%)	8 (2.8%)	2 (0.7%)	1 (0.3%)	22 (1.7%)
***P. ovale***	0	1 (0.5%)	0	0	0	1 (0.1%)
**Total**	231 (79 [34.2%])*	196 (23[11.7%])*	286 (116[40.6%])*	270 (115 [42.6])*	301 (46 [15.3])*	1284 (379[29.5%])*

*Locally acquired cases.

The *P. knowlesi/P. malariae* cases in Selangor rose from 21 cases (9.1% of 231) in 2009 to 25 cases (12.8% of 196) in 2010, then peaked at 43 cases (15% of 286) in 2011 and 41 cases both in 2012 (15.2% of 270) and 2013 (13.6% of 301).

### *Plasmodium knowlesi/Plasmodium malariae*cases from 2009–2013 in Hulu Selangor district

Among the nine districts in Selangor, the Hulu Selangor district charted the most cumulative number of *P. knowlesi/P. malariae* cases consisting of 100 (58.48% of 171) cases over the five years (i.e., 2009–2013) period. There were 4 cases of *P. knowlesi/P. malariae* notified in 2009, 5 in 2010, then an increase of 7 fold to 35 cases in 2011, declining to 30 cases in 2012 and 26 cases in 2013.

Of these 100 *P. knowlesi/P. malariae* cases, 91 (91%) cases involved male versus 9 (9%) female cases (Table 2). Cases of *P. knowlesi/P. malariae* were more predominant among the young adults (ages 20–39 years; 67 cases; 67%) followed by those above 40 years (23 cases; 23%). Based on nationality, 51 cases (51%) were Malaysians followed by Indonesians (35 cases; 35%). The majority of the infected people were involved in occupation related to agriculture and forestry (51; 51%). No death was recorded in all these cases.

**Table 2:** **Characteristics of** ***Plasmodium knowlesi/Plasmodium malariae*** **cases from 2009 till 2013 in Hulu Selangor district (Total cases = 100)**

Variable	2009	2010	2011	2012	2013	Total
	(Total case = 4)	(Total case = 5)	(Total case = 35)	(Total case = 30)	(Total case = 26)	
***Gender***						
Male	4	5	33	25	24	**91**
Female	0	0	2	5	2	**9**
***Age*** **(years)**						
<1-9	0	0	0	0	0	**0**
10-19	1	0	2	4	3	**10**
20-29	2	2	6	9	7	**26**
30-39	0	3	19	10	9	**41**
40-49	0	0	4	2	5	**11**
>50	1	0	4	5	2	**12**
***Nationality***						
Malaysian	3	0	17	15	16	**51**
Indonesian	0	3	14	10	8	**35**
Myanmarese	0	1	1	1	0	**3**
Cambodian	0	0	2	0	0	**2**
Filipino	0	0	1	0	0	**1**
Nepalese	0	0	0	1	2	**3**
Indian	1	0	0	2	0	**3**
Bangladeshi	0	1	0	1	0	**2**
***Occupation***						
Agriculture	0	3	22	13	10	**48**
Construction	0	1	0	4	2	**7**
Forestry	0	0	2	1	0	**3**
Industry	1	1	1	3	5	**11**
Security/Soldier/Police	1	0	3	3	2	**9**
Civil servant	0	0	0	1	0	**1**
Hotel/Tourism	1	0	1	0	1	**3**
Self employed/Housewife/Maid	0	0	5	2	3	**10**
Student	1	0	1	3	3	**8**
***Outcome***						
Alive	4	5	35	30	26	**100**
Dead	0	0	0	0	0	**0**

### Species composition of *Anopheles*mosquitoes

Five hundred and thirty five mosquitoes belonging to 14 species were obtained during the study as shown in Table 3. *Anopheles maculatus* was the predominant species (49.5%) followed by *An. letifer* (13.1%) and *An. introlatus* (11.6%)*.* However, *An. maculatus* and *An. letifer* were collected in large numbers only once in a particular site. A*nopheles latens* was the only other mosquito belonging to the Leucosphyrus group besides *An. introlatus*.

**Table 3:** ***Anopheles*** **species collected from Hulu Selangor district between 2012 to 2013**

Mosquito species	Numbers collected	Percentage (%)
*An. donaldi*	2	0.4
*An. hyrcanus gr.*	26	4.9
*An. introlatus*	62	11.6
*An. karwari*	39	7.3
*An. latens*	2	0.4
*An. letifer*	70	13.1
*An. maculatus*	265	49.5
*An. peditaniatus*	2	0.4
*An. philippinensis*	10	1.9
*An. separatus*	15	2.8
*An. sinensis*	19	3.6
*An. tesselatus*	1	0.2
*An. umbrosus gr*	2	0.4
Total	535	100.00

**Figure 1:**
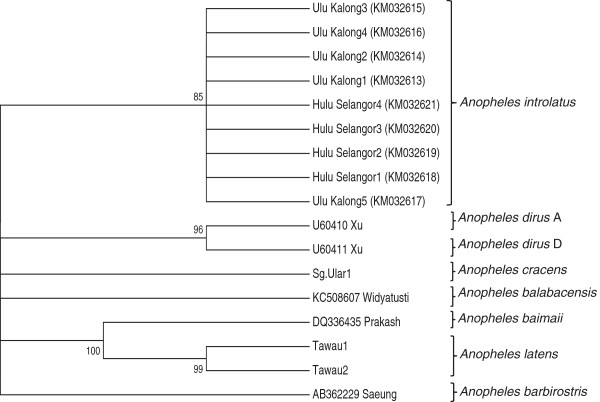
**Phylogenetic tree based on the ITS2 gene produced by the neighbor-joining method.** Figures on the branches are bootstrap percentages based on 1000 replicates.

**Figure 2:**
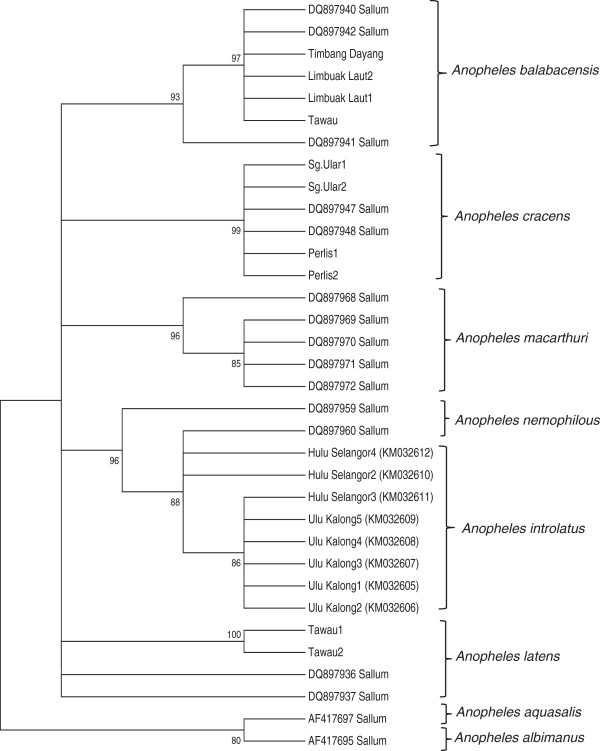
**Phylogenetic tree based on the CO 1 genes produced by the neighbor-joining method.** Figures on the branches are bootstrap percentages based on 1000 replicates.

Molecular analysis confirmed the species of Leucosphyrus group to be *An. introlatus*. Phylogenetic analysis inferred from the NJ method showed that the ITS2 region of nine adult *An. introlatus* (KM032613-KM032621) caught from various areas where knowlesi malaria cases occurred formed a monophyletic clade supported by a strong bootstrap value (Figure 1). Further phylogenetic analysis based on the CO1 gene found that six isolates (KM032605-KM032609; KM032611) also clustered in a monophyletic clade (Figure 2).

### Vector efficiency of *Anopheles introlatus*

The efficiency of a vector depends on how long it lives and how frequently it bites humans. The gonotrophic cycle of *An. introlatus* was taken as three days (this was based on observation in the laboratory where the blood fed mosquito laid eggs on the 3rd day after a blood meal). The daily survival rate [45], life expectancy [46] and vectorial capacity [47] of *An. introlatus* are shown in Table 4. The parous rate of *An. introlatus* was 65.5%. This showed that more than half of the mosquitoes were potentially dangerous. Of these, 25% of the *An. introlatus* would be expected to live the 10 days for the *P. knowlesi* sporozoites to be formed. Those surviving the 10 days would have a further life expectancy of 7.2 days. However, the vectorial capacity was only 1.94 since the man biting rate was low.

**Table 4:** **Parous rate, life expectancy and vectorial capacity of** ***Anopheles introlatus***

Number dissected	55
Parous rate	65.5
Probability of survival (p)^1^	0.87
P^10^ (%)	25
p^10^/-log_e_ p (days)^2^	7.2
Vectorial capacity ^3^	1.94

^1^Thus the daily probability of survival (p) was taken as ^3^√P (P = percentage parous) [45].

p^10^ percentage of population expected to live long enough to become infective with an extrinsic cycle of 10 days for *P. knowlesi.*

^2^life expectancy [46].

^3^vectorial capacity VC = ma^2^p^n^/-log^e^p [47].

**Figure 3:**
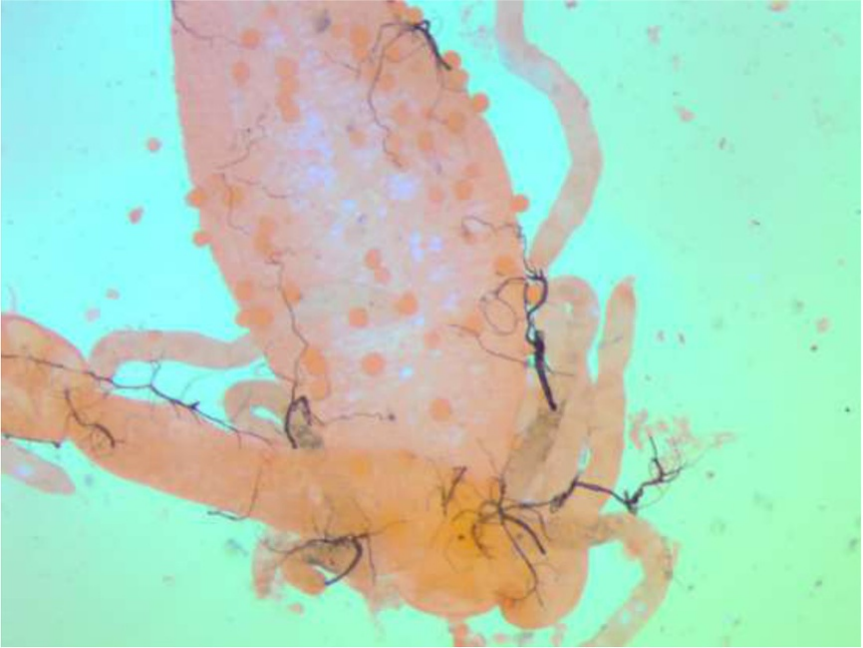
**Midgut of the** ***An. introlatus*** **positive with 56 oocysts.**

**Figure 4:**
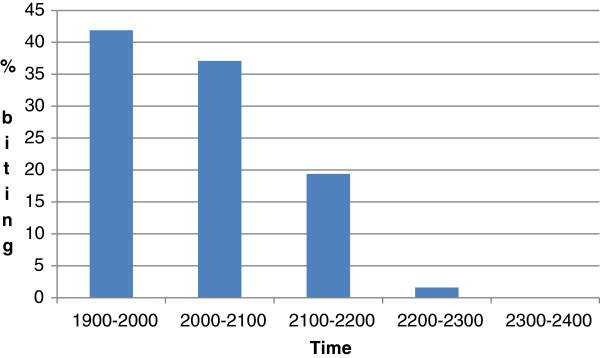
**Biting times of** ***Anopheles introlatus*** **in Hulu Selangor district.**

However, the most important index of transmission is the number of mosquitoes infected with sporozoites. In this study none of the mosquitoes were infected with sporozoites. Only two *An. introlatus* were positive for oocysts. One was found positive with 56 occysts in the midgut (Figure 3). The PCR results showed it was positive for *P. knowlesi*. The other had only two oocysts and PCR failed to detect any species. The infected rate was 1.82 (1/55 dissected). In addition, *An. introlatus* was found biting in the early part of the night. The peak biting rate was from 1900 to 2100 as shown in Figure 4. Most of the *An. introlatus* was obtained biting humans in the forest.

### Temporal and spatial analysis of *Plasmodium knowlesi/Plasmodium malariae*cases

**Figure 5:**
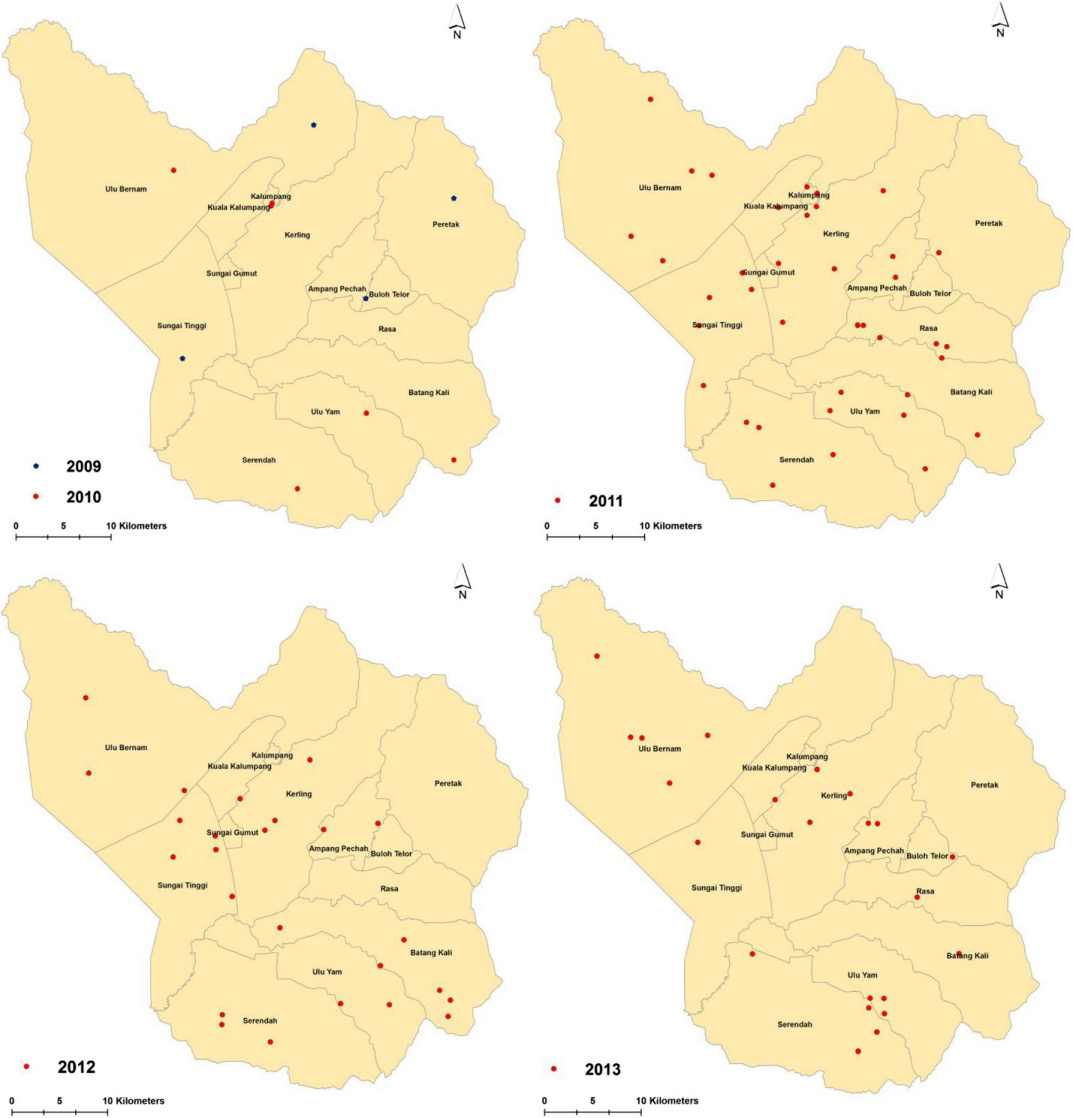
**Temporal distribution pattern of** ***P. knowlesi/P. malariae*** **cases in Hulu Selangor sub-district, Selangor by year (2009–2013).** The map demonstrates a clear spatial pattern of *P. knowlesi/P. malariae* cases that was mostly spread in all sub-districts in Hulu Selangor.

The ANN analysis of *P. knowlesi/P. malariae* cases demonstrated several dissemination patterns on a yearly basis (Figure 5). Generally, the distribution pattern of *P. knowlesi/P. malariae* cases from 2009 to 2013 in Hulu Selangor sub-districts were statistically clustered with an R value of less than 1 (R = 0.77; Z = -4.55; p < 0.01). The *P. knowlesi/P. malariae* cases in 2012 were clustered in certain sub-districts with an R value of 0.82 (Z = -186; p < 0.1). In contrast, a dispersed distribution pattern was observed for cases in 2011 (R = 1.16; z = 198; p < 0.05), 2010 (R = 1.40; z = 187; p < 0.1) and 2009 (R = 4.27; z = 12.52; p < 0.001), while a random dissemination pattern was reported for *P. knowlesi/P. malariae* cases that occurred in 2013 (R = 102; z = 021; p = 1).

**Figure 6:**
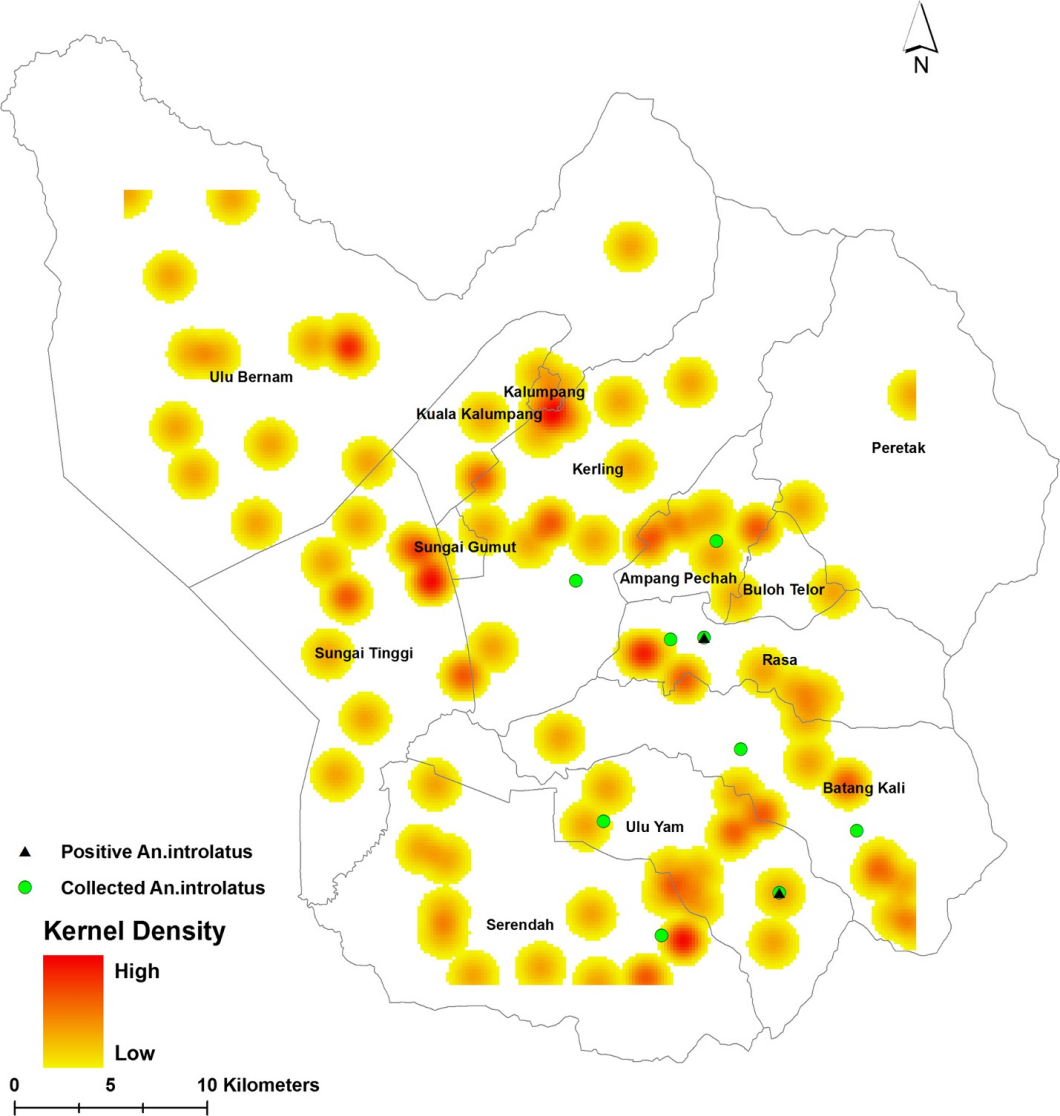
**Hot spot location of** ***P. knowlesi/P. malariae*** **cases in Hulu Selangor sub-districts assessed using Kernel Density.**

The prevalence of *P. knowlesi/P. malariae* cases reported in 2009 to 2013 was further analyzed using Kernel Density to identify high risk areas at sub-district level (Figure 6). The *P. knowlesi* density map can assist in precisely identifying the location, spatial extent and intensity of *P. knowlesi/P. malariae* case hotspots. The result showed that the red colored area was identified as a hotspot area with high *P. knowlesi/P. malariae* cases. In contrast, the location with yellow color indicates low density of hot spots areas. The Kernel Density map demonstrated the most affected area with *P. knowlesi/P. malariae* cases. Generally, most of the hot spot locations of *P. knowlesi/P. malariae* cases were mostly spread in all sub-districts of Hulu Selangor. It was demonstrated that the highest density hot spot of *P. knowlesi/P. malariae* cases were located in between the border of Sungai Tinggi and Sungai Gemut and the border of Kalumpang and Kerling. Likewise, Rasa and Serendah sub-districts were also identified as an area with high *P. knowlesi/P. malariae* cases.

## Discussion

In the state of Selangor, only 29.5% of malaria cases were locally acquired from 2009 until 2013. Throughout this period, although malaria cases were predominantly caused by *P. vivax* (71.3%) followed by *P. falciparum* (13.6%), cases of *P. knowlesi/P. malariae* (13.3%) is trailing very close to falciparum infections and showing an upward trend. The single grouping of *P. knowlesi/P. malariae* was adopted as the primary method of diagnosis of these malaria cases and was based on microscopy examination of peripheral blood smears with a small subset of specimens confirmed via PCR. In order to reduce death cases and heighten recognition and management of potentially fatal malaria species, Rajahram *et al.* [48]. suggested the need for microscopically diagnosed *P. malariae* to be reported as *P. knowlesi* in Sabah. Thus, it is highly recommended that future studies incorporate molecular techniques to decipher confirmed cases of *P. knowlesi* in order to arrive to a better conclusion with regards to the trend of *P. knowlesi* cases.

Now that knowlesi malaria is occurring in many areas, it is important to determine the vectors so that appropriate control measures can be instituted. *Anopheles maculatus* was the predominant mosquito obtained in this study and it is also the known vector of human malaria in peninsular Malaysia [26]. However, one needs to be careful in the interpretation of the data. Currently, only the Leucosphyrus group of mosquitoes have been incriminated as vectors of simian malaria [49]. Studies in Kuala Lipis also showed that *An. cracens,* which belongs to the Leucosphyrus group was the vector. Although *An. maculatus* was obtained in that study, none were positive [25, 34]. Laboratory studies have also shown that it is only the Leucosphyrus group of mosquitoes that are vectors [49]. The other mosquitoes for instance, *An. freeborni* can produce sporozoites but it cannot invade the salivary glands [50].

In the 1960s studies carried out in the lowland swamp forest using monkey bait and human bait traps, obtained four species of mosquitoes (not belonging to the Leucosphyrus group) namely *An. donaldi*, *An. letifer*, *An. umbrosus*, and *An. roperi,* which were positive for sporozoites. These sporozoites were inoculated into rhesus monkeys and long-tailed macaques but none turned positive [51]. Four decades later studies have shown that only Leucosphyrus group of mosquitoes are able to transmit simian malaria [25, 32–34].

In the present study, only *An. introlatus* was positive for oocysts. The mosquito must be able to develop sporozoites before it can be considered a vector. However, it is highly possible that *An. introlatus* will be able to produce sporozoites. The oocysts were not melanised and with such large numbers of oocysts being present in one mosquito, it may be the vector in the area. Moreover, Kernel Density analysis showed that *P. knowlesi* hotspot areas overlapped with areas where the infected *An. introlatus* was discovered. This further strengthens the hypothesis that *An. introlatus* is the vector for the *P. knowlesi* cases in the Hulu Selangor district. This is the first report of *An. introlatus* in Hulu Selangor. It has been previously reported from Gombak, Selangor, peninsular Malaysia [51], Thailand and Indonesia [38].

Studies in the 1960s have also incriminated *An. introlatus* as a vector of *P. cynomolgi* and *P. fieldi* [52]. *Anopheles introlatus* is known to be found only in small numbers in jungle areas [26]. In studies in the forest canopy using human and monkey bait traps at canopy level (15 meters), it was found that more *An. introlatus* were attracted to monkeys at canopy level compared to humans [51]. The *An. introlatus* in this study may be biting humans by chance and that could also be one reason why the number of cases was not very high as compared to Sabah and Sarawak in Malaysian Borneo. It has been demonstrated that *An. introlatus* are exophilic and early biters, which makes vector control very challenging. Thus, the current vector control measures like indoor residual spraying and insecticide treated nets will not be able break the chain of transmission and control the vectors. More innovative approaches will be required.

From this study, it is obvious that since mosquito populations are found only in small numbers, it is not possible to determine the vectors by carrying out surveys only once or twice in each site. A large number of field workers will have to be distributed throughout the sites in order to select the best sites for the collection of mosquitoes. This is also reflected in an earlier study [53], where they were not able to incriminate the vector. It is also known that the Asia Pacific Region has the highest number of dominant vector species for human malaria compared to other regions and is important to determine the correct species for control activities to succeed [54]. Thus, it shows that more rigorous sampling has to be carried out to determine the vectors of simian malaria. It has been noted that entomological studies are scanty and more areas need to be covered [55, 56]. However, one of the limitations for these studies is the involvement of humans for collection of mosquitoes.

With deforestation, the long tail macaques (*Macaca fascicularis*) have come to the forest fringes and perhaps these mosquitoes may have followed the macaques and colonized forest fringes. It is also possible that *An. introlatus* may be attracted more to monkeys than humans as shown in studies carried out four decades ago [51]. Studies should be carried out with monkey baited traps to determine if these mosquitoes are attracted to monkeys. Due to logistic problems, it was not possible to set up monkey baited traps.

**Figure 7:**
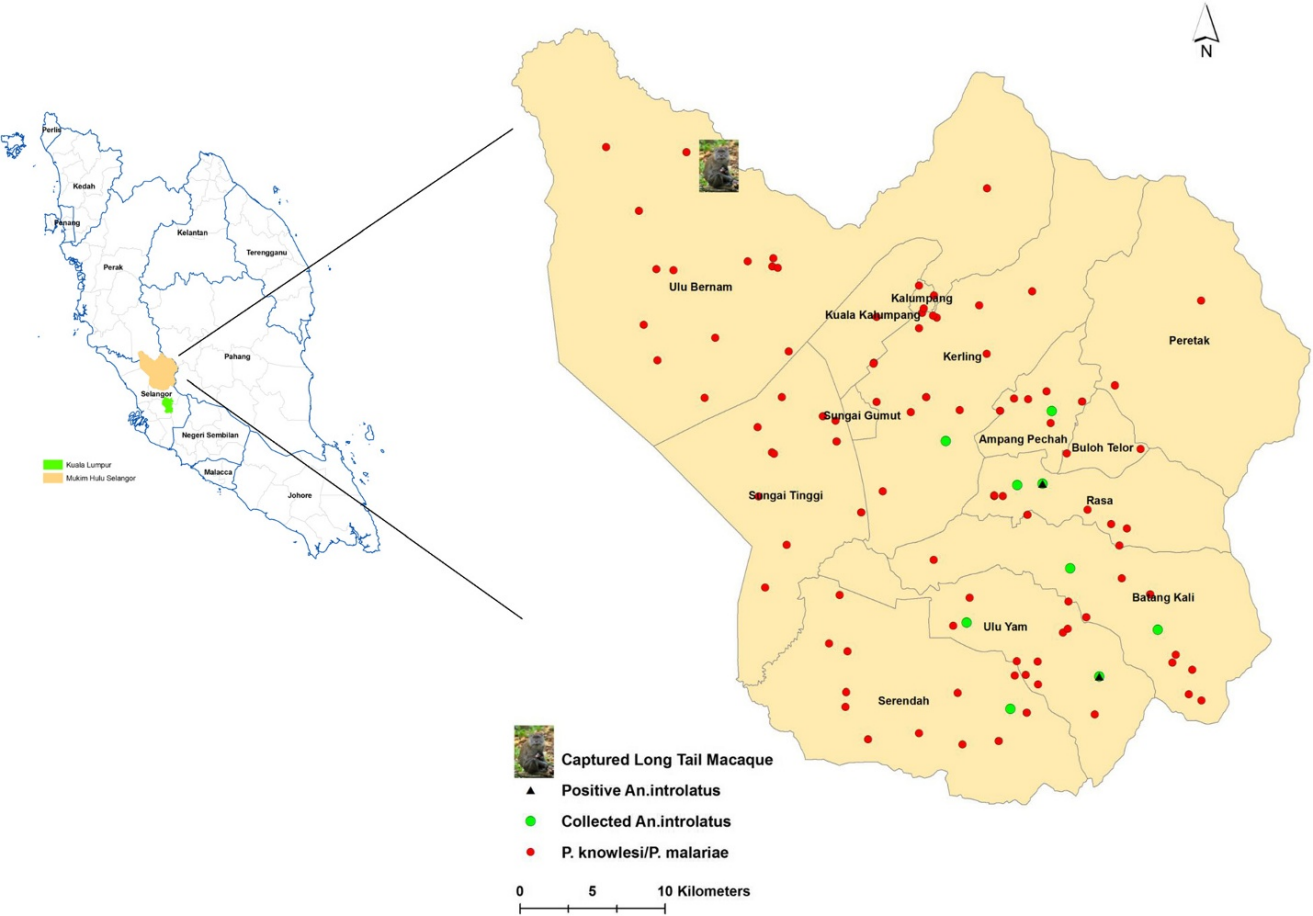
**Spatial dissemination pattern of overall** ***P. knowlesi/P. malariae*** **cases,** ***An. introlatus*** **mosquitoes and long tail macaque in Hulu Selangor sub-districts.**

In 2013, 15 *M. fascicularis* were trapped at Ulu Bernam, Kuala Kubu Bharu (Figure 7). Of this total, none were positive with *P. knowlesi.* However, the presence of other simian *Plasmodium* such as *P. inui*, *P. coatneyi*, *P. fieldi* and *P cynomolgi* were high and a majority had mixed infections (unpublished data, personal communication with Reuben Sharma). Although the macaques were not infected with *P. knowlesi*, the high infection rates (53.3% - 80%) with other simian malaria is of public health concern.

This is especially so as a recent article has highlighted that besides *P. knowlesi,* another simian species, *P. cynomolgi* has also been found to infect humans naturally. This first known case was observed in a 39-year-old woman from a malaria-free area in Terengganu (east coast state of Malaysia). Although the initial diagnosis was *P. malariae/P. knowlesi* by microscopy, subsequent PCR assay with sequencing analysis confirmed that the patient was infected with *P. cynomolgi* [57]. As *P. cynomolgi* is morphologically indistinguishable from *P. vivax*, the true cases may be under-diagnosed [57]. In view of this limitation, it is crucial that novel molecular-based diagnostic tools are developed particularly those with multiplex systems to include the identification of these simian malaria species.

In terms of elimination of malaria it refers to only human malaria [58]. However, here is a situation where human malaria has been reduced or is in the process of being eliminated and simultaneously knowlesi malaria is on the rise in affecting humans. Thus, from the public health point of view, it is difficult to explain to the community that malaria has been eliminated when cases are still occurring in larger numbers than before.

Thus for elimination of malaria to be successful, the vector(s) need to be determined. With changing landscape and ecology, there will be changes to vector distribution and thus new maps for vector distribution is needed so as to enable an efficient control strategy.

As illustrated by the epidemiological data, the knowlesi malaria is also to some extent related to the migrant workers working in the forest fringe late at night tapping rubber. These people live in the forest fringe and the houses are just a bare minimum with temporary walls. *Anopheles introlatus* mosquitoes have been collected close by to their dwellings. In Southeast Asia, it has also been documented that the migrant population living in forest fringe villages, and also the ethnic minority groups in villages surrounded by forest are at risk of malaria [59]. In Thailand, Myanmar, eastern Bangladesh, western Cambodia, southern Laos and Vietnam, there is usually a close association between forests and the *An. dirus* complex. This vector may also be present in fruit orchards, but at lower density than in forests [60–62].

In peninsular Malaysia, it was always the *An. maculatus* that was the predominant vector transmitting malaria to the aborigine population in the forest area [37]. However, the situation has changed and now like the neighbouring countries the Leucosphyrus group mosquitoes are involved in malaria transmission [25, 34]. Thus, vector ecology and transmission patterns of knowlesi malaria present a unique challenge for vector control management, which is pivotal for malaria elimination.

In addition, based on the results of this study, utilization of tools such as GIS analysis should be highly recommended as a surveillance tool particularly in areas where there are high incidence rates. Spatial analysis has highlighted that in 2013, cases were highly clustered in certain sub-districts, however in 2012, a random dissemination pattern was observed. Having this knowledge at hand will enable control and prevention strategies to be more targeted, efficient and more cost effective. Finally, it also shows that the utilization of molecular techniques is crucial in the detection of the various malaria species so as to avoid mortality due to malaria species misidentification.

## Conclusion

From this study, it can be concluded that more effort is warranted in research and development for control of knowlesi malaria cases. Unless more information is obtained on the vectors as well as macaques involved in the transmission, it will be difficult to plan any effective control strategies. The utilization of modern analytical tools such as GIS is crucial in estimating hotspot areas for targeted control strategies. With the elimination of human malaria, cases of knowlesi malaria will be on the increase in coming years as evident from this study. Thus, there is a greater need for multi disciplinary research to combat simian malaria affecting humans.
